# Effects of Obesity on Pregnancy and Fetomaternal Outcome

**DOI:** 10.7759/cureus.94358

**Published:** 2025-10-11

**Authors:** Faryal Rehman, Summaya Asmat, Noreen Mehsud, Laila Ishtiaq, Kinza Shakeel, Maimoona Ali, Laiba LNU, Rida Ahmad, Abdur Rafah, Muhammad Suffyan

**Affiliations:** 1 Department of Obstetrics and Gynaecology, Hayatabad Medical Complex, Peshawar, PAK; 2 Department of Obstetrics and Gynaecology, Bacha Khan Medical College, Mardan, PAK; 3 Department of Obstetrics and Gynaecology, Mardan Medical Complex, Mardan, PAK; 4 Department of Obstetrics and Gynaecology, Lady Reading Hospital, Peshawar, PAK; 5 Department of Obstetrics and Gynaecology, Letterkenny University Hospital, Co, Donegal, IRL; 6 Department of Obstetrics and Gynaecology, Combined Military Hospital, Peshawar, PAK; 7 Department of Obstetrics and Gynaecology, Khyber Teaching Hospital, Peshawar, PAK; 8 Department of Medicine and Surgery, Bacha Khan Medical College, Mardan, PAK; 9 Department of Medicine and Surgery, Hayatabad Medical Complex, Peshawar, PAK; 10 Department of Paediatrics and Neonatology, City Hospital, Multan, PAK

**Keywords:** cesarean section, fetal complications, intensive care unit, macrosomia, maternal obesity, nicu admission, pregnancy outcomes

## Abstract

Introduction: A growing public health risk linked to unfavorable pregnancy outcomes is maternal obesity. With an emphasis on the incidence and seriousness of difficulties in pregnancies complicated by obesity, this study aims to assess the impact of obesity on the health of both the mother and the fetus.

Methodology: 178 pregnant women with a BMI of 30 kg/m² participated in this 12-month prospective comparative cohort research at the Hayatabad Medical Complex, Peshawar. Group A (BMI 30-34.9 kg/m²; n = 96, 53.9%) and Group B (BMI ≥35 kg/m²; n = 82, 46.1%) were the two groups into which the participants were split. Maternal outcomes (gestational hypertension, preeclampsia, gestational diabetes, cesarean delivery, postpartum hemorrhage, and wound infection) and fetal outcomes (birth weight, macrosomia, Apgar score, neonatal intensive care unit (NICU) admission, and stillbirth) were documented according to Royal College of Obstetricians and Gynaecologists (RCOG) guidelines. Chi-square and independent t-tests were used in the statistical analysis, which was conducted using SPSS version 26.0. A p-value of less than 0.05 was deemed statistically significant.

Results: Group B had significantly higher rates of gestational hypertension 26 (31.7%) vs. 18 (18.8%), preeclampsia 18 (21.9%) vs. 10 (10.4%), cesarean section 51 (62.2%) vs. 39 (40.6%), and wound infection 11 (13.4%) vs. 4 (4.2%) compared to Group A (*p* < 0.05). Neonatal outcomes were also worse in Group B, including macrosomia 19 (23.2%) vs. 7 (7.3%), low Apgar scores <7 at 5 minutes 14 (17.1%) vs. 6 (6.3%), and NICU admissions 24 (29.3%) vs. 12 (12.5%). Composite fetomaternal morbidity was significantly more common in Group B 35 (42.7%) vs. 20 (20.8%).

Conclusion: Higher degrees of maternal obesity are strongly associated with increased maternal and neonatal complications. Early identification and specialized prenatal care for obese women are essential to minimize risks and improve outcomes.

## Introduction

The problem of obesity, defined by the World Health Organization (WHO) as a BMI ≥30 kg/m², has become a global health concern and a major contributor to pregnancy complications between a mother and her child [1]. Overweight is defined as a BMI ≥25 kg/m² and obesity as a BMI ≥30 kg/m² in adults, while age must be considered when defining overweight and obesity in children [2]. The prevalence of obesity has risen significantly among women of reproductive age in the past few decades, largely driven by sedentary lifestyles, high-fat diets, and socioeconomic changes [3]. This trend is particularly worrying given the physiological and metabolic alterations associated with pregnancy, which are further exacerbated by excessive adiposity [4-6].

Obesity in maternal figures is known to be complex to many adverse effects, to the mother and the fetus as well [5]. Obese women have a higher chance of getting gestational hypertension and preeclampsia, Gestational diabetes mellitus (GDM), and thromboembolism, as well as cesarean births [6]. These complications not only add morbidity and mortality among mothers but also impose a huge burden on health systems [7]. In addition to that, obese patients usually require longer labor, and are more prone to a medical upforce or a surgery termination becoming riskier during a procedure and lengthening the recovery time [8].

Obesity impacts the mother only, but the impacts raise significant risks to fetal development and outcomes during infancy [9]. Obese mothers are more likely to experience macrosomia, shoulder dystocia, birth trauma, increased admission into neonatal critical care units, and low Apgar scores [10]. Maternal obesity also contributes to an intergenerational cycle of obesity and associated health problems, as maternal obesity has long-term consequences among the children as they start developing a higher propensity toward childhood obesity, metabolic syndrome, and type 2 diabetes when they grow older [11].

Although some connections had already been made between obesity and poor pregnancy outcomes, limited and conflicting information persists in terms of low- and middle-income countries, including Pakistan, wherein sociocultural and dietary-related factors and accessibility of healthcare potentially leave a unique mark on the outcomes. [12]. Moreover, it does not find any region-specific research being done to investigate the degree to which obesity can influence fetomaternal health, especially in the tertiary care facilities [13]. This study will be able to fill the data gap in the country by evaluating the effect of maternal obesity on pregnancy progression and fetomaternal outcomes in a tertiary care hospital.

## Materials and methods

Study design and setting

This prospective comparative cohort study was conducted at the Department of Obstetrics and Gynecology of Hayatabad Medical Complex (HMC), Peshawar, over a period of 12 months from May 2023 to May 2024. The hospital serves a diverse patient population from both urban and semi-rural regions, thereby allowing adequate representation of women from varied sociodemographic backgrounds. The primary aim of the study was to evaluate the impact of maternal obesity on maternal and fetal health outcomes, with specific attention to the frequency, severity, and overall pattern of complications.

Sample size calculation

The sample size was determined using the World Health Organization (WHO) sample size calculator. The calculation was based on an anticipated prevalence of adverse fetomaternal outcomes of 25.2% among obese pregnancies, as reported in previous literature [14], with a 95% confidence level, a 5% margin of error, and 80% study power. This yielded a minimum required sample size of 163 participants. To allow for potential nonresponse, attrition, or incomplete data, the sample was increased to 178 pregnant women. This adjustment ensured that the study maintained sufficient statistical power to detect clinically meaningful differences, particularly for outcomes with moderate prevalence.

Inclusion and exclusion criteria

Participants were recruited consecutively from antenatal clinics and inpatient admissions. Screening was performed during their first antenatal visit or at the time of hospital admission, and eligibility was confirmed against predefined criteria. Written informed consent was obtained from all participants after they were fully informed about the objectives, procedures, potential risks, and expected benefits of the study in a language they could understand.

The inclusion criteria comprised pregnant women aged 18 to 40 years with a body mass index (BMI) of at least 30 kg/m² at their first prenatal visit and carrying a singleton pregnancy of at least 20 weeks’ gestation. BMI was calculated using the conventional formula of weight in kilograms divided by the square of height in meters (kg/m²). Women with multiple gestations, pre-existing diabetes mellitus, chronic hypertension, cardiovascular disease, renal disease, or established congenital fetal anomalies were excluded from the study. These exclusions were made deliberately to reduce confounding and to ensure that observed outcomes could be more confidently attributed to maternal obesity rather than other comorbidities.

Data collection tool and standard protocols

The primary tool for data collection was a structured questionnaire designed in accordance with the standard protocols of antenatal assessment prescribed by the Royal College of Obstetricians and Gynaecologists (RCOG) guidelines. This instrument was used to systematically document demographic characteristics, obstetric history, BMI, antenatal complications, delivery records, and fetal outcomes. Maternal and neonatal assessments were carried out following the established recommendations of the RCOG and the WHO, ensuring consistency and reliability in data recording [15, 16]. To further strengthen the internal validity of the study, all obstetric and neonatal evaluations were performed by trained healthcare professionals who adhered to uniform procedures.

Grouping and comparison

The stratification of the participants was done into two groups: women who had Class I obesity (BMI 30-34.9 kg/m²) belonged to Group A, whereas women with Class II and III obesity (BMI ≥35 kg/m²) were classified in Group B. The two groups were used to compare and determine whether there is an increase in maternal obesity and any relationship with fetomaternal outcomes. The stratification enabled it to be understood better how different levels of obesity actually can impact the occurrence and intensity of pregnancy and newborn complications. For analytical purposes, the study population was stratified into two groups based on maternal BMI. Women with Class I obesity (BMI 30-34.9 kg/m²) were categorized into Group A, while those with Class II and III obesity (BMI ≥35 kg/m²) were categorized into Group B. This stratification enabled a direct comparison of outcomes across increasing levels of obesity, thereby allowing the assessment of whether the severity of obesity was proportionately associated with worsening fetomaternal outcomes.

Outcome measures

The primary outcomes of interest were maternal and fetal complications attributable to obesity during pregnancy. Maternal outcomes included gestational hypertension, preeclampsia, prolonged labor, GDM, mode of delivery (spontaneous vaginal, instrumental, or cesarean), postpartum hemorrhage, wound infection, and maternal intensive care unit (ICU) admission. Fetal outcomes assessed were birth weight, macrosomia, intrauterine growth restriction (IUGR), preterm birth, low Apgar scores at five minutes, neonatal intensive care unit (NICU) admissions, stillbirth, and perinatal mortality. All outcomes were carefully documented and classified according to standardized RCOG guidelines to maintain uniformity.

Statistical analysis

All data were entered into and analyzed using SPSS version 26.0. Continuous variables such as maternal age, BMI, and birth weight were summarized using means and standard deviations, while categorical variables such as gestational hypertension, preeclampsia, and NICU admission were presented as frequencies and percentages. Comparisons between the two groups were carried out using independent t-tests for continuous variables and chi-square tests for categorical variables. A p-value of less than 0.05 was considered statistically significant. To further account for confounding, multivariable logistic regression was performed for each binary outcome to estimate adjusted odds ratios (aORs) with 95% confidence intervals, adjusting for maternal age, parity, booking status, and gestational age at delivery. For rare outcomes such as stillbirth, Firth’s penalized logistic regression was employed to minimize small-sample bias. Sensitivity analyses were also conducted using BMI as a continuous variable and propensity-score weighting, which provided an additional layer of robustness in validating the findings.

Ethical considerations

The study protocol was reviewed and approved by the Institutional Research and Ethical Board of Hayatabad Medical Complex, Peshawar (approval number: 813/OG/HMC/2023). All participants provided written informed consent prior to inclusion. Confidentiality of personal information was assured, and participants were informed of their right to withdraw from the study at any stage without compromising the quality of their medical care.

## Results

There was no statistically significant difference between the mean mother ages in Group A and Group B, which were 29.4 ± 4.7 and 30.2 ± 5.1 years, respectively (t = 1.08, p = 0.237), as shown in Table 1. This difference was very significant (t = 17.39, p < 0.001), with the mean BMI of Group B being much higher (37.9 ± 2.6 kg/m²) than that of Group A (32.4 ± 1.3 kg/m²). Group A and Group B included 41 (42.7%) and 37 (45.1%) primigravida patients, respectively, but Group B had 55 (57.3%) and 45 (54.9%) multigravida patients; this difference was not statistically significant (χ² = 0.12, p = 0.729). These results lessen the possibility of confounding by showing that the two groups were similar in terms of age and parity. The significantly higher BMI in Group B confirms the intended stratification and sets the foundation for comparing subsequent outcomes.

**Table 1:** Maternal baseline characteristics. Values presented as Mean ± SD or N (%). *A p-value < 0.05 was considered significant.

Variable	Group A (n = 96)	Group B (n = 82)	Statistical Test	p‑value
Mean age (years)	29.4 ± 4.7	30.2 ± 5.1	t = 1.08	0.237
Mean BMI (kg/m²)	32.4 ± 1.3	37.9 ± 2.6	t = 17.39	<0.001 *
Primigravida	41 (42.7 %)	37 (45.1 %)	χ² = 0.12	0.729
Multigravida	55 (57.3 %)	45 (54.9 %)	χ² = 0.12	0.729

**Figure 1:**
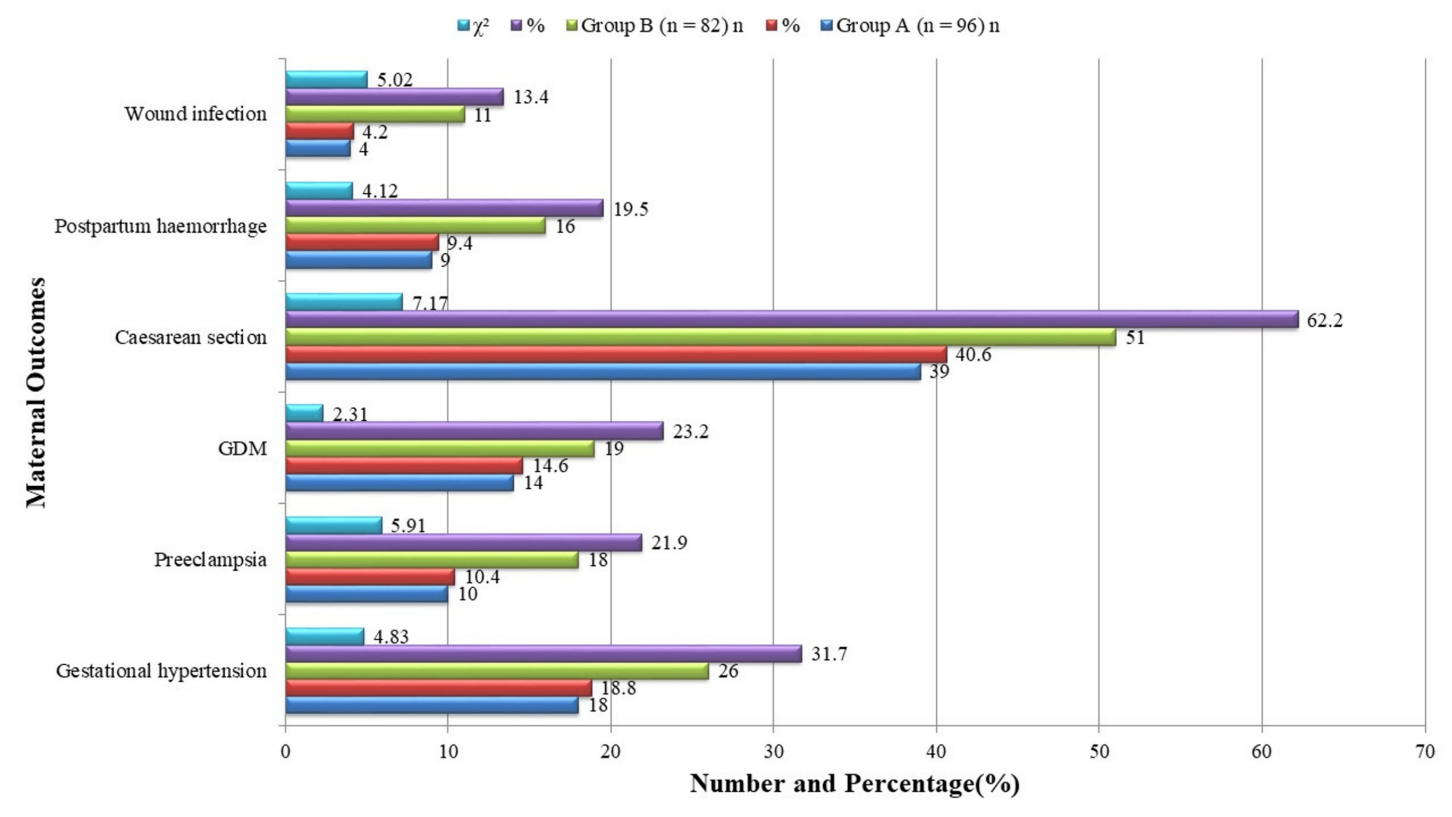
Maternal outcomes.

Figure 1 illustrates several adverse maternal outcomes, with a higher prevalence in the more obese group. Eighteen women (18.8%) in Group A and 26 women (31.7%) in Group B were found to have gestational hypertension; this difference was statistically significant (χ² = 4.83, p = 0.028). Ten women (10.4%) in Group A, and eighteen (21.9%) in Group B developed preeclampsia (χ² = 5.91, p = 0.015). The percentage of women who had cesarean sections was significantly greater in Group B, with 51 (62.2%) versus 39 (40.6%) in Group A (χ² = 7.17, p = 0.007). Sixteen cases (19.5%) in Group B and nine cases (9.4%) in Group A had postpartum hemorrhage (χ² = 4.12, p = 0.042), and 11 women (13.4%) in Group B had wound infection compared to 4 (4.2%) in Group A (χ² = 5.02, p = 0.025). Despite being more common in Group B 19 (23.2%) compared to Group A 14 (14.6%), GDM did not reach statistical significance (χ² = 2.31, p = 0.118). These findings demonstrate a robust correlation between rising maternal BMI and unfavorable maternal outcomes, particularly complications from hypertension and surgery.

As shown in Table 2, labor and delivery outcomes were more unfavorable in the higher obesity group. Group B had a substantially lower mean gestational age at delivery (37.1 ± 1.4 weeks) than Group A (38.2 ± 1.3 weeks), with a highly significant difference (t = 5.40, p < 0.001). Thirteen (13.5%) of the women in Group A, and twenty-one (25.6%) of the women in Group B experienced prolonged labor (χ² = 4.42, p = 0.035). Additionally, Group B 13 had a substantially greater rate of failed induction (15.9%) compared to Group A 6 (6.3%) (χ² = 4.31, p = 0.038). In Group A, the rate of surgical vaginal delivery was 7 (7.3%), while in Group B, it was 5 (6.1%) (χ² = 0.10, p = 0.748). These results imply that early births, more challenging labor, and higher rates of labor interventions are linked to severe maternal obesity. This emphasizes the importance of individualized labor management in obese pregnant women.

**Table 2:** Labor and delivery characteristics. Values presented as Mean ± SD or n (%). *A p-value <0.05 was considered significant.

Variable	Group A (n = 96)	Group B (n = 82)	Statistical Test	OR (95% CI)	p-value
Mean gestational age at delivery (weeks)	38.2 ± 1.3	37.1 ± 1.4	t = 5.40	Mean diff = 1.1 (95% CI 0.7–1.5)	<0.001 *
Prolonged labor	13 (13.5%)	21 (25.6%)	χ² = 4.42	2.2 (1.1–4.7)	0.035 *
Failed induction	6 (6.3%)	13 (15.9%)	χ² = 4.31	2.8 (1.0–7.7)	0.038 *
Operative vaginal delivery	7 (7.3%)	5 (6.1%)	χ² = 0.10	0.8 (0.2–2.5)	0.748

As shown in Table 3, Group B's mean birth weight was noticeably greater (3410 ± 410 g) compared to Group A (3120 ± 380 g) (t = 4.86, p < 0.001). Macrosomia was observed in 19 (23.2%) neonates in Group B versus 7 (7.3%) in Group A (χ² = 9.35, p = 0.002). Six (6.3%) and fourteen (17.1%) newborns in Group B and Group A had low Apgar scores (<7 at 5 minutes) (χ² = 5.02, p = 0.025). According to χ² = 7.84, p = 0.005, NICU admissions were similarly substantially greater in Group B 24 (29.3%) than in Group A 12 (12.5%). There were three (3.7%) stillbirths in Group B and none in Group A; nevertheless, this difference was close to but fell short of statistical significance (χ² = 3.65, p = 0.056). These results strongly imply that there is a link between maternal obesity and higher rates of newborn problems and morbidity. Enhanced fetal surveillance and NICU readiness are warranted in obese pregnancies.

**Table 3:** Fetal and neonatal outcomes. Values presented as Mean ± SD or N (%). *A p-value < 0.05 was considered significant.

Outcome	Group A (n = 96)	Group B (n = 82)	Statistical Test	OR (95% CI)	p-value
Mean birth weight (g)	3120 ± 380	3410 ± 410	t = 4.86	Mean diff = 290 (95% CI 170–410)	<0.001 *
Macrosomia > 4000 g	7 (7.3%)	19 (23.2%)	χ² = 9.35	3.8 (1.5–9.4)	0.002 *
Apgar <7 (5 min)	6 (6.3%)	14 (17.1%)	χ² = 5.02	3.1 (1.1–8.5)	0.025 *
NICU admission	12 (12.5%)	24 (29.3%)	χ² = 7.84	2.9 (1.3–6.3)	0.005 *
Stillbirth	0 (0%)	3 (3.7%)	χ² = 3.65	—	0.056

**Figure 2:**
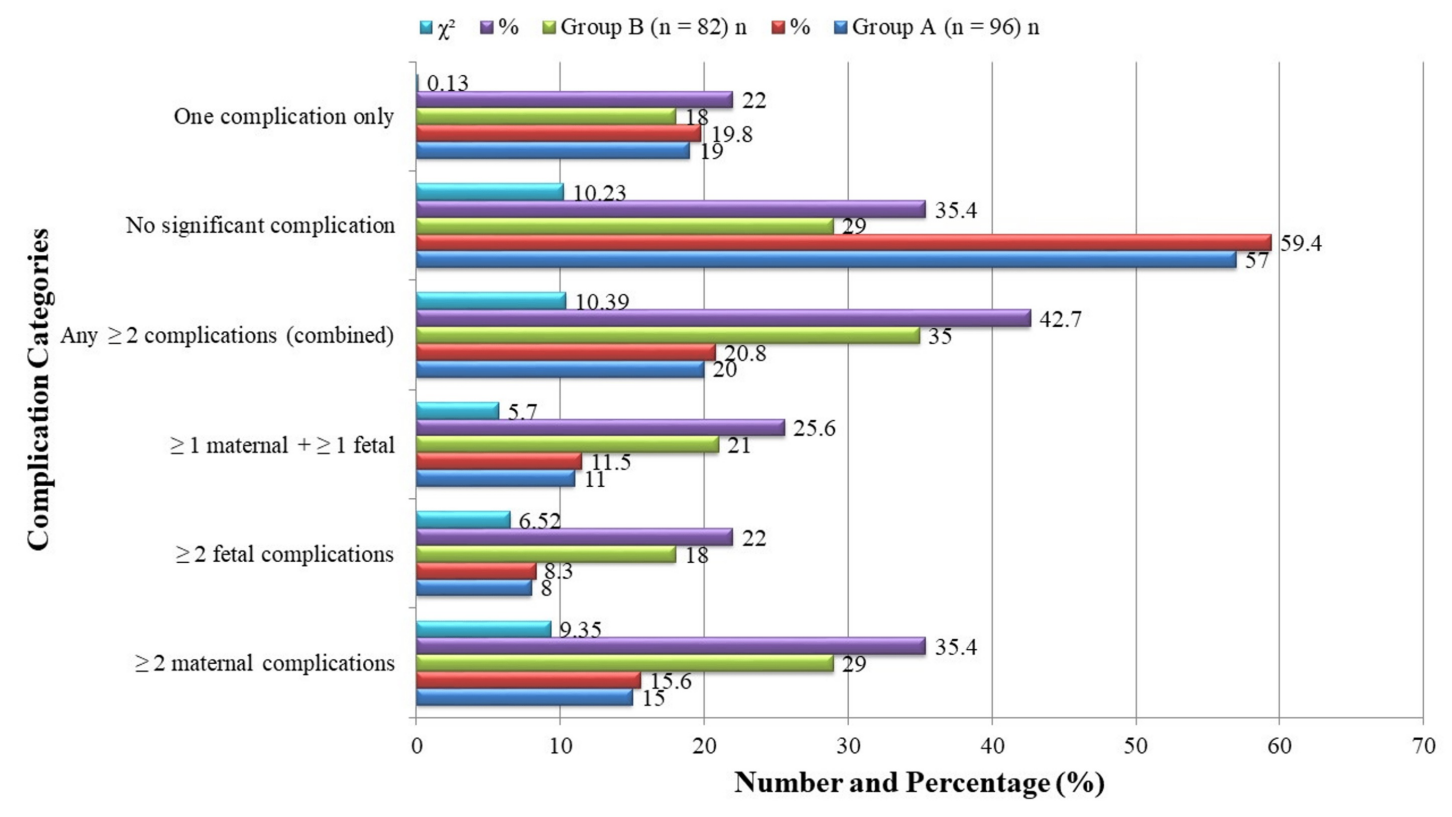
Expanded composite fetomaternal morbidity profile.

As illustrated in Figure 2, a significantly higher proportion of women in Group B experienced ≥2 maternal complications 29, 35.4%) compared to Group A 15, 15.6%) (χ² = 9.35, p = 0.002). Similarly, ≥2 fetal complications occurred in 18 (22.0%) Group B patients versus 8 (8.3%) in Group A (χ² = 6.52, p = 0.011). The combination of at least one maternal and one fetal complication was also more frequent in Group B (21 (25.6%)) than in Group A 11 (11.5%) (χ² = 5.70, p = 0.017). The overall proportion of women with any ≥2 complications (maternal, fetal, or both) was significantly higher in Group B (35 (42.7%)) versus Group A 20 (20.8%) (χ² = 10.39, p = 0.001). Only 29 (35.4%) women in Group B had no significant complications, compared to 57 (59.4%) in Group A (χ² = 10.23, p = 0.001), whereas the percentage of women with only one complication was statistically similar in both groups (χ² = 0.13, p = 0.716). This composite analysis reinforces the cumulative risk burden of obesity in pregnancy, necessitating more vigilant prenatal care in this population.

## Discussion

This study compared women with Class I obesity (BMI 30-34.9 kg/m²) to those with Class II and III obesity (BMI ≥35 kg/m²) in order to find out how maternal obesity affects pregnancy and fetomaternal outcomes. The findings showed that poor maternal outcomes, such as gestational hypertension, preeclampsia, cesarean delivery, postpartum hemorrhage, and wound infections, were substantially more likely to occur in women with higher BMIs. Similar effects were shown in fetal outcomes, with the higher BMI group experiencing greater rates of macrosomia, low Apgar scores, NICU admissions, and stillbirths. Additionally, composite fetomaternal morbidity, particularly the presence of multiple simultaneous complications, was significantly more common in women with BMI ≥35 kg/m².

This study validates that the effect of increasing maternal adiposity is direct and quantifiable on pregnancy outcomes and safety for both the mother and the child. Our findings, particularly the association of high maternal BMI with hypertensive disorders of pregnancy, are consistent with international literature [16-18]. For instance, a cross-sectional study from Kuwait reported that GDM was significantly associated with higher maternal age and prepregnancy BMI, and was linked to adverse outcomes such as increased rates of cesarean section delivery and fetal macrosomia [17]. Similarly, a retrospective study from Vienna demonstrated that maternal obesity in women with GDM was closely related to treatment intensity, with obese mothers requiring higher doses of insulin or metformin, and being at greater risk of cesarean delivery and delivering large-for-gestational-age infants [18]. Taken together, these findings align with our results and emphasize that maternal adiposity not only predisposes women to hypertensive disorders but also contributes to gestational diabetes, the need for more intensive pharmacological interventions, and poor neonatal outcomes. This strengthens the evidence that maternal obesity is a multidimensional risk factor, adversely influencing both maternal and fetal health through interrelated metabolic and obstetric pathways. Higher cesarean section rates in the case of obese women have also always been pointed out in previous studies, probably because of increased rates of unsuccessful induction, macrosomia, and labor dystocia [18].

The higher incidence of postpartum hemorrhage and wound infection observed in our study is consistent with previous evidence, which attributes these complications to poor wound healing, altered inflammatory responses, and increased obstetric risk in obese individuals. A study from South Africa further highlighted that women with obesity had significantly higher rates of postpartum hemorrhage, failed induction, fetal anomalies, and unscheduled prenatal care visits. Moreover, neonates of obese mothers were more likely to require neonatal unit admission, and the overall costs of care were substantially higher in this group compared with normal-weight women, underscoring the added burden obesity imposes on resource-constrained healthcare systems [19]. Neonatal outcomes in our cohort, particularly low Apgar scores and the need for NICU admission, also mirror prior evidence linking maternal obesity to obesity-related metabolic disturbances, prolonged labor, and impaired neonatal adaptation. A comprehensive review additionally reported that while weight reduction through bariatric surgery may reduce obesity-related complications, pregnancies occurring soon after surgery carry risks such as low birth weight, small-for-gestational-age infants, and micronutrient deficiencies, highlighting the complex interplay between maternal nutritional status, obesity, and perinatal outcomes [20].

Furthermore, the elevated risk of macrosomia and stillbirth observed in our study reflects established physiological mechanisms such as insulin resistance and placental dysfunction, which are prevalent in obese pregnancies [21]. Evidence from a prospective cohort study conducted in Lahore similarly demonstrated that obese women had significantly higher rates of instrumental vaginal delivery, cesarean delivery, postpartum hemorrhage, macrosomia, poor Apgar scores, and NICU admissions compared to non-obese controls, underscoring the reproducibility of these associations in South Asian populations [21]. In addition, a retrospective study evaluating women with hyperglycemia diagnosed in early pregnancy found that this group exhibited a more severe insulin resistance phenotype, with adverse outcomes including large-for-gestational-age and small-for-gestational-age neonates, neonatal hypoglycemia, and hyperbilirubinemia [22]. Taken together, these findings reinforce the evidence that obesity and related metabolic disturbances exert a compounded effect on maternal and neonatal health, while also emphasizing the cumulative burden of overlapping complications in obese pregnancies.

This study's findings have significant clinical and public health ramifications. The increased burden of operative delivery, hypertensive disorders, and neonatal complications in obese pregnant women places a significant strain on healthcare systems, particularly in resource-limited settings [23]. The higher frequency of NICU admissions and maternal surgical interventions observed in this study suggests increased hospital stay durations, healthcare costs, and emotional stress for families. These results underscore the necessity for integrated antenatal care models that include nutritional counseling, weight monitoring, and early screening for metabolic complications [24]. In addition, the observed clustering of multiple complications in severely obese women highlights the need for multidisciplinary management, involving obstetricians, nutritionists, endocrinologists, and neonatologists [25]. Preventive strategies aimed at weight optimization before conception could substantially reduce the prevalence of high-risk pregnancies and improve overall obstetric outcomes [26]. Therefore, addressing maternal obesity should be a national priority within maternal health policies and programs [27].

Limitations and future suggestions

This study has certain limitations despite the robustness of the results. First, as it was conducted at a single tertiary care facility, the findings may not be generalizable to rural or low-resource settings with different demographic and healthcare profiles. Second, obesity was defined solely by BMI, without incorporating other anthropometric or metabolic indicators such as waist-to-hip ratio, visceral adiposity, or biochemical markers, which may provide a more comprehensive risk assessment. Third, some outcomes, such as stillbirth, were relatively infrequent, which limited the statistical power to detect significant associations.

Although we attempted to minimize confounding by ensuring similarity between groups in baseline variables such as age and parity, residual confounding from unmeasured factors (e.g., physical activity, dietary patterns, socioeconomic status, or genetic predisposition) could not be fully excluded. Future studies should therefore include multivariate models with broader sets of covariates to strengthen causal inference.

To improve external validity and refine risk prediction, multicenter studies with larger and more diverse cohorts are recommended. Additionally, future research should explore the role of metabolic and inflammatory markers in mediating obesity-related pregnancy risks. From a clinical perspective, evaluating interventions such as preconception weight optimization programs, tailored antenatal surveillance protocols, and individualized labor management strategies for obese women will be essential to improving maternal and neonatal outcomes.

## Conclusions

This study emphasizes the substantial influence of maternal obesity on unfavorable pregnancy and fetomaternal outcomes, especially at BMI ≥35 kg/m². Hypertensive disorders, cesarean deliveries, postpartum problems, and poor neonatal outcomes, such as macrosomia and NICU admissions, were more common among women in the greater obese group. The presence of multiple, overlapping complications was also notably higher in this group. These findings emphasize the need for targeted prenatal monitoring, early risk stratification, and comprehensive management strategies for obese pregnant women to reduce maternal and neonatal morbidity.
